# Mangiferin Decreases Plasma Free Fatty Acids through Promoting Its Catabolism in Liver by Activation of AMPK

**DOI:** 10.1371/journal.pone.0030782

**Published:** 2012-01-23

**Authors:** Yucun Niu, Songtao Li, Lixin Na, Rennan Feng, Liyan Liu, Ying Li, Changhao Sun

**Affiliations:** Department of Nutrition and Food Hygiene, Public Health College, Harbin Medical University, Harbin, Heilongjiang, People's Republic of China; Boston University, United States of America

## Abstract

Mangiferin has been shown to have the effect of improving dyslipidemia. Plasma free fatty acids (FFA) are closely associated with blood lipid metabolism as well as many diseases including metabolic syndrome. This study is to investigate whether mangiferin has effects on FFA metabolism in hyperlipidemic rats. Wistar rats were fed a high-fat diet and administered mangiferin simultaneously for 6 weeks. Mangiferin (50, 100, 150 mg/kg BW) decreased dose-dependently FFA and triglycerides (TG) levels in plasma, and their accumulations in liver, but increased the β-hydroxybutyrate levels in both plasma and liver of hyperlipidemic rats. HepG2 cells were treated with oleic acid (OA, 0.2 mmol/L) to simulate the condition of high level of plasma FFA in vitro, and were treated with different concentrations of mangiferin simultaneously for 24 h. We found that mangiferin significantly increased FFA uptake, significantly decreased intracellular FFA and TG accumulations in HepG2 cells. Mangiferin significantly increased AMP-activated protein kinase (AMPK) phosphorylation and its downstream proteins involved in fatty acid translocase (CD36) and carnitine palmitoyltransferase 1 (CPT1), but significantly decreased acyl-CoA: diacylgycerol acyltransferase 2 (DGAT2) expression and acetyl-CoA carboxylase (ACC) activity by increasing its phosphorylation level in both in vivo and in vitro studies. Furthermore, these effects were reversed by Compound C, an AMPK inhibitor in HepG2 cells. For upstream of AMPK, mangiferin increased AMP/ATP ratio, but had no effect on LKB1 phosphorylation. In conclusion, mangiferin decreased plasma FFA levels through promoting FFA uptake and oxidation, inhibiting FFA and TG accumulations by regulating the key enzymes expression in liver through AMPK pathway. Therefore, mangiferin is a possible beneficial natural compound for metabolic syndrome by improving FFA metabolism.

## Introduction

A large body of evidence suggests that elevated plasma free fatty acids (FFA) are a risk factor for metabolic syndrome, including insulin resistance, atherogenic dyslipidemia and type 2 diabetes [1], [2]. Elevated FFA can inhibit the anti-lipolytic action of insulin, interfere with insulin signaling, and inhibit insulin-stimulated glucose uptake and glycogen synthesis [3], [4]. Therefore, pharmacological agents that could effectively lower plasma FFA are likely to have a significant effect on improving metabolic syndrome [5].

The liver is an important site of FFA removal from the blood [6]. At rest, about 80% of plasma FFA is mainly transported to the liver where they are either oxidized to generate energy in the form of ATP, or re-esterified for storage as triglycerides (TG) [7], [8]. Not surprisingly, increased plasma FFA levels may result in intracellular accumulation of lipid metabolites in the liver, leading to fatty liver, liver insulin resistance and type 2 diabetes. Therefore, one potential strategy for improving metabolic syndrome is not only to reduce the level of plasma FFA, but also to promote FFA uptake and oxidation instead of accumulation of intracellular TG in the liver.

Mangiferin is a xanthone glucoside and exists in many kinds of food and folk medicines such as mangoes and *Anemarrhena asphodeloides* rhizomes. Mangiferin has many beneficial biological activities, including anti-inflammatory, anti-oxidant and anti-diabetic effects [9], [10]. Recent studies showed that mangiferin significantly reduced the level of plasma TG in diabetic animals [11], [12]. Plasma TG must first be hydrolyzed into FFA and glycerol, and then is taken up and utilized by tissues in the form of FFA [13], [14]. Therefore, plasma TG metabolism is associated closely with plasma FFA. However, there are no reports on whether mangiferin can affect FFA levels or FFA metabolism in liver. Therefore, the aim of our study was to investigate the effects of mangiferin on plasma FFA levels, FFA metabolism and its possible mechanisms. Using both *in vivo* and *in vitro* models, we found mangiferin was able to decrease the levels of plasma FFA, promote FFA catabolism, and inhibit the synthesis of FFA and TG in liver through AMPK signaling pathway.

## Results

### Mangferin had no toxic effect in rats and HepG2 cells

Rats were fed with different concentrations of mangiferin (0, 100, 200, 400 mg/kg BW) for 30 days. There were no significant effects on body weight, blood cell counting, hemoglobin, urea nitrogen, creatinine, blood glucose, total cholesterol, triglycerides, aspartate transaminase and alanine transaminase activity (Table S1). This indicated that mangiferin was nontoxic up to the dose of 400 mg/kg BW and was used in different doses for further studies. At the same time, after 24 h mangiferin treatment, cytotoxicity was observed when the concentration of mangiferin reached 400 µmol/L (data not shown). Therefore, mangiferin concentration was safe within 12.5–100 µmol/L/L in the present study.

### Mangiferin decreased plasma FFA and TG levels, and inhibited liver FFA and TG accumulations in hyperlipidemic rat

At the end of 6 weeks, the rats in hyperlipidemia group had higher levels of TC (*P*<0.05), TG (*P*<0.01) and FFA (*P*<0.01) in both plasma and liver tissue compared with rats in normal control (Table 1), indicating that the high-fat diet induced successfully hyperlipidemic rats model with triglycerides, FFA and cholesterol accumulations in the liver. Fenofibrate (a positive drug) and all doses of mangiferin supplementation significantly decreased plasma FFA level (*P*<0.05), and fenofibrate and mangiferin at 100 or 150 mg/kg BW significantly decreased plasma TG level (*P*<0.01), and liver FFA (*P*<0.01) and TG (*P*<0.01) levels in hyperlipidemic rats. In addition, fenofibrate and high dose of mangiferin may decrease the weights of abdominal and epididymal fat pads in hyperlipidemic rats (Table 1). Mangiferin had a tendency of lowering TC in all the doses, but did not reach the statistical significance.

**Table 1:** Effect of mangiferin on fasting metabolic variables at 6 weeks in hyperlipemic rats.

Measurement	Control	Hyperlipidemia	Hyperlipidemia+fenofibrate(100 mg/kg BW)	Hyperlipidemia+mangiferin(50 mg/kg BW)	Hyperlipidemia+mangiferin(100 mg/kg BW)	Hyperlipidemia+mangiferin(150 mg/kg BW)
Body weight (g)	340.3±24.9	378.5±32.6##	368.1±34.7	371.2±28.3	369.7±35.1	361.4±36.7
Food intake (g/day)	26.56±2.17	33.25±3.98##	31.97±3.01	32.23±2.75	34.02±2.70	32.13±2.64
Liver weight (g)	8.43±1.21	8.97±1.33	8.79±1.25	8.88±1.24	8.80±1.12	8.71±1.08
Abdominal fat (g)	10.23±1.28	15.15±1.42##	11.16±1.27**	14.43±1.39	13.79±1.41*	12.81±1.34*
Epididymal fat (g)	8.78±1.03	10.23±1.24#	8.98±0.98*	10.02±1.15	9.46±1.02	9.04±1.12*
Plasma TC (mmol/L)	3.70±0.47	4.26±0.61#	3.87±0.32*	4.15±0.47	4.02±0.39	4.08±0.37
Plasma TG (mmol/L)	0.89±0.12	1.46±0.18##	0.96±0.19**	1.37±0.22	1.24±0.16**	1.05±0.10**
Plasma FFA (mg/dl)	484.3±37.5	587.6±41.4##	501.8±36.2**	541.6±30.8*	527.8±33.9**	493.5±38.1**
Plasma β-hydroxybutyrate (µmol/L)	93.2±8.47	64.8±6.91##	87.5±9.17**	70.3±7.10	83.5±9.13**	90.6±10.01**
Liver TC (µmol/g of liver)	5.80±0.34	7.18±0.37##	6.51±0.32*	7.02±0.30	6.98±0.38	6.87±0.36
Liver TG (µmol/g of liver)	4.06±0.22	5.13±0.32##	4.43±0.37**	4.89±0.33	4.42±0.30**	4.12±0.39**
Liver FFA (µmol/g of liver)	84.8±14.4	153.5±27.7##	104.6±22.4**	139.1±21.9	118.6±21.3**	97.6±23.8**
Liver β-hydroxybutyrate(µmol/g of liver)	7.23±0.71	4.23±0.57##	6.87±0.83**	4.88±0.50*	5.87±0.61**	6.43±0.78**

Data are means ± SD (n = 10),

# *P*<0.05

## *P*<0.01 indicate statistically significant differences when compared with control group.

*P<0.05 and

**P<0.01 indicate statistically significant differences when compared with hyperlipidemia group.

### Mangiferin regulated the key enzymes of FFA metabolism and TG synthesis in liver of hyperlipidemic rat

**Figure 1:**
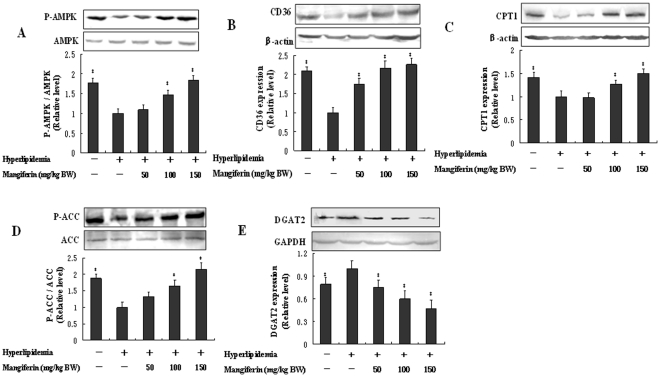
Effects of mangiferin on the proteins expression of FFA metabolism including AMPK, CD36, CPT1, ACC and DGAT2 in liver of hyperlipidemic rats. Wistar rats were divided randomly into five groups (n = 10 per group): control group (fed an AIN-93G diet); hyperlipidemia group (fed a high-fat diet); mangiferin-supplemented groups, fed the high-fat diet and different doses of mangiferin (50, 100, 150 mg/kg BW/d). The experiment lasted for 6 weeks, and the liver was taken for western blot analysis. (A) AMPK phosphorylation level. (B) CD36 expression on cell membrane. (C) CPT1 expression in mitochondrion. (D) ACC level and activity. (E) DGAT2 expression. * P<0.05 compared with hyperlipidemic group.

AMPK is a key mediator in the control of intracellular lipid metabolism, including the uptake, synthesis and oxidation of fatty acid in liver [20]. Mangiferin significantly activated hepatic AMPKα subunit (*P*<0.05, Figure 1A) by phosphorylating AMPKα at the Thr-172 residue in hyperlipidemic rats. The downstream proteins of AMPK including CD36, CPT1, ACC and DGAT2 were also affected by mangiferin treatment. The expression of CD36 protein, which is involved in regulating FFA uptake [21], was increased significantly by mangiferin (*P*<0.05, Figure 1B). CPT-1 and ACC are the rate-limiting enzymes for FFA β-oxidation and synthesis, respectively, in liver [22], [23]. Mangiferin increased significantly the expression of CPT-1 (*P<0.05*, Figure 1C) and the level of ACC phosphorylation (*P<0.05*, Figure 1D) which has been shown to reduce the activity of the enzyme in a dose-dependent manner. Additionally, β-hydroxybutyrate, a main intermediate metabolite of FFA oxidation in liver [24], was increased significantly by mangiferin in both plasma and liver of hyperlipidemic rats, which indicating that FFA oxidation might be increased. DGAT2 is a key enzyme that catalyzes the final step and rate-limiting reaction in TG synthesis [25]. Mangiferin decreased DGAT2 expression significantly (*P<0.05*, Figure 1E) in liver of hyperlipidemic rats. These results indicate collectively that mangiferin promotes the FFA uptake and oxidation, and inhibits FFA and TG synthesis by regulating the key enzymes expression in liver of hyperlipidemic rats.

### Mangiferin increased FFA uptake, decreased intracellular FFA and TG accumulations in HepG2 cells

**Figure 2:**
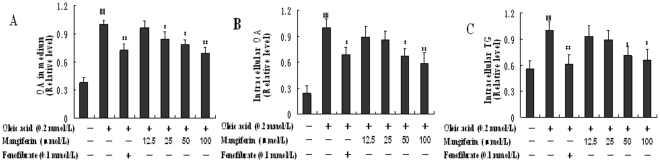
Effects of mangiferin on OA in medium, intracellular OA and intracellular TG in HepG2 cells. HepG2 cells were incubated with 0.2 mmol/L OA only or with different concentrations of mangiferin (12.5, 25, 50, 100 µmol/L) or fenofibrate (100 µmol/L) simultaneously for 24 h. The concentrations of OA in medium (A) and intracellular OA (B) after the incubation were determined by GC-MS. The intracellular TG (C) mass was quantified by the enzymatic methods using a TG test kit. Data are presented as means ± SD (n = 3). ^#^ *P*<0.05 and ^##^ *P*<0.01 compared with the normal control. ^*^ *P*<0.05 and ^**^ *P*<0.01 compared with only OA stimulation group.

HepG2 cells were treated with OA (0.2 mmol/L) in order to simulate the condition of high level of plasma FFA in animal experiment. Mangiferin significantly decreased the OA concentration of cell culture medium in a dose-dependent manner, and fenofibrate (positive control) at 100 µmol/L also significantly decreased the OA concentrations (*P*<0.01, Figure 2A). OA uptake by HepG2 cells was calculated by the concentration of OA remaining in the medium after treatment. Compared with only OA stimulation group, mangiferin at 25, 50 and 100 µmol/L increased the OA uptake by 22.4%, 24.2% and 32.4% respectively. In addition, mangiferin at 50, 100 µmol/L and fenofibrate significantly decreased the content of intracellular OA (*P*<0.05, Figure 2B) and TG (*P*<0.05, Figure 2C) in HepG2 cells. Further, the actual numbers used to generate the relative results of Figure 2 are listed in supplementary Table S2. These results indicate that mangiferin could decrease the level of FFA in medium and inhibit TG accumulation in hepatic cells.

### Mangiferin regulated the key enzymes of FFA metabolism and TG synthesis in HepG2 cells

**Figure 3:**
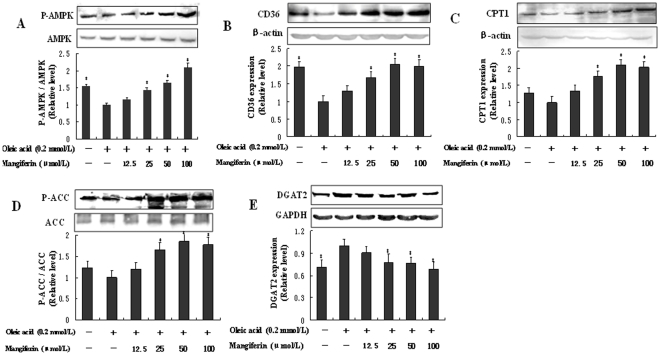
HepG2 cells were incubated with 0.2 mmol/L OA only or with different concentrations of mangiferin (12.5, 25, 50, 100 µmol/L) simultaneously for 24 h. Proteins were isolated from the cell lysates and analyzed by western blot analysis for AMPK (A), CD36 (B), CPT1 (C), ACC (D) and DGAT (E) expressions. The experiments were repeated 3 times. Data are presented as means ± SD (n = 3). ^*^ *P*<0.05 compared with only OA stimulation group.

To further confirm the mechanism of mangiferin on improving FFA metabolism and inhibiting TG synthesis *in vitro*, we determined the protein levels of AMPK, CD36, CPT1, ACC and DGAT2 in HepG2 cells by mangiferin supplementation. Mangiferin significantly increased the phosphorylation levels of AMPK and ACC (*P*<0.05, Figure 3A, 3D), the protein levels of CD36 and CPT1 (*P*<0.05, Figure 3B, 3C), but decreased significantly the protein level of DGAT2 (*P*<0.05, Figure 3E). These data suggest that mangiferin could promote FFA uptake and oxidation, inhibit FFA and TG synthesis by regulating the key enzymes expression in HepG2 cells.

### AMPK was involved in the effect of mangiferin on FFA metabolism and TG synthesis in HepG2 cells

**Figure 4:**
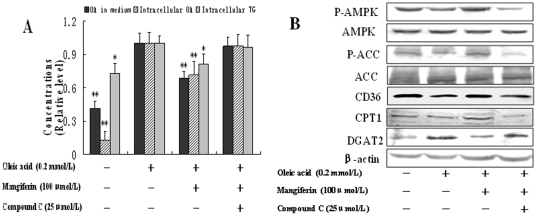
Effects of compound C on mangiferin induced FFA uptake, intracellular FFA, TG and FFA metabolism proteins expression in HepG2 cells. HepG2 cells were pretreated 1 h with compound C, an AMPK inhibitor, and then treated with 100 µmol/L mangiferin and 0.2 mmol/L OA for 24 h. OA in medium (To assess the uptake of OA) (A), intracellular OA (A), intracellular TG (A) and proteins expression of FFA metabolism including AMPK (B), ACC (B), CD36 (B), CPT1 (B) and DGAT2 (B) were determined by western blot method. The experiments were repeated 3 times. Data are presented as means ± SD (n = 3). ^*^ *P*<0.05 and ^**^ *P*<0.01 compared with only OA stimulation group.

Compound C, an AMPK inhibitor, abolished almost completely the activation of AMPKα by mangiferin. The effects of mangiferin on OA uptake, intracellular OA and TG (Figure 4A) contents, as well as the expression of AMPK downstream proteins, including CD36, CPT1, P-ACC and DGAT2 were also suppressed significantly after compound C treatment (*P*<0.05, Figure 4B). These results strongly suggest that mangiferin exerts its effect on lowering plasma FFA level, promoting FFA uptake and oxidation, inhibiting FFA and TG synthesis via the AMPK signaling pathway in liver.

### AMPK was phosphorylated and activated by AMP/ATP but not LKB1 in mangiferin signaling in HepG2 cells

**Figure 5:**
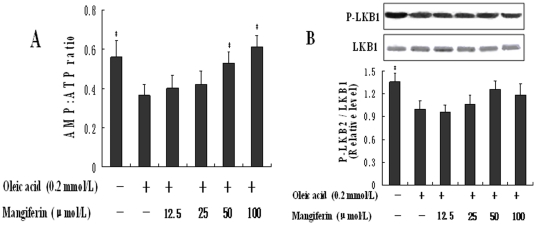
Effects of mangiferin on the ratio of AMP to ATP and LKB1 protein expression in HepG2 cells. HepG2 cells were incubated to 0.2 mmol/L oleic acid only or with different concentrations of mangiferin (12.5, 25, 50, 100 µmol/L) simultaneously for 24 h. The ratio of AMP to ATP was detected by HPLC (A). The LKB1 protein expression was carried out by western blot analysis (B). The experiments were repeated 3 times. Data are presented as means ± SD (n = 3). ^*^ *P*<0.05 compared with only OA stimulation group.

To investigate how mangiferin activates AMPK, we determined the effect of mangiferin on upstream of AMPK, including the ratio of AMP to ATP and LKB1 activity and protein expression [26]. Mangiferin increased the ratio of AMP to ATP in a dose-dependent manner in HepG2 cells (*P*<0.05, Figure 5A). However, neither the content of LKB1 nor the ratio of p-LKB1/LKB1 was altered significantly by mangiferin (Figure 5B). These results indicate that mangiferin activates AMPK by increasing the ratio of AMP to ATP instead of LKB1 in present study.

## Discussion

There is considerable public and scientific interest in the various beneficial biological activities of mangiferin, including antioxidant, anti-inflammatory and anti-diabetic effects [9], [10]. However, there are few studies about the effect of mangiferin on dyslipidemia. Miura and Huang et al [11], [27] reported only that mangiferin may decrease the levels of blood TC and TG [11], [12], and that the mechanism is not yet clear. FFA is a major component of blood lipids and plays a key role in regulating blood lipid levels, especially in triglycerides metabolism [28]. In addition, elevated plasma FFA is a risk factor for metabolic syndrome, which can lead to hyperlipidemia, fatty liver and insulin resistance [2], [29]. However, there is no report about the effect of mangiferin on FFA levels and FFA metabolism.

*In vivo*, we used a high-fat diet to induce hyperlipidemia model in rats. The rats in hyperlipidemia groups had higher body weight, plasma TC, TG and FFA levels and liver TC, TG and FFA levels compared to rats in normal control group. These results indicated that the hyperlipidemic rats model with triglycerides and cholesterol accumulation in the liver was induced successfully by the high-fat diet. In order to study further the mechanism improving FFA metabolism *in vitro*, HepG2 cells were treated with OA (0.2 mmol/L) to simulate the condition of high plasma levels of FFA in animal experiment. As a result, 0.2 mmol/L OA increased significantly the intracellular TG content in liver cells, a condition that is observed often when plasma FFA is elevated in human and animal studies. Fenofibrate, a hypotriglyceride drug as positive control agent, can reduce plasma FFA and hepatic TG [30]. In our study, fenofibrate significantly increased FFA uptake, and decreased intracellular FFA and TG in HepG2 cells, indicating that using OA-treated HepG2 cells to study the effect of mangiferin on FFA metabolism is feasible.

We have confirmed that the doses of mangiferin used in this study are safe based on the toxicity experiment of 30-day feeding test and MTT assay both *in vivo* and in *vitro*. Our study showed for the first time that mangiferin decreased significantly plasma FFA levels in hyperlipidemic rats and FFA levels in culture medium of HepG2 cells. It is well known that plasma FFA is mainly transported into the liver at rest. CD36 is the rate-limiting enzyme in high-affinity peripheral FFA uptake in the liver [21], [31]. We have found that CD36 is involved in the increased FFA uptake supplemented with mangiferin in liver of hyperlipidemic rats and in OA induced HepG2 cells, a conclusion that is supported by the increase of CD36 protein expression. Mangiferin increased FFA uptake, decreased the liver FFA levels in hyperlipidemic rats and in HepG2 cells, which indicates an increased FFA utilization in liver. In the liver, FFA is converted intracellularly into fatty-acyl-CoA to produce energy in the form of long-chain fatty-acyl-CoAs (LCACoAs) through β-oxidation or esterification into TG [8]. β-hydroxybutyrate (ketone body) is the main alternative energy substrate to glucose, and is generated by hepatic fatty acid oxidation in liver when plasma glucose and insulin are low [32]. Mangiferin increased the levels of β-hydroxybutyrate both in blood and liver in our study, indicating that mangiferin could increase FFA oxidation. In addition, CPT-1 is the rate-limiting enzyme for LCACoAs transport from the cytoplasm into the mitochondria in FFA β-oxidation. The activity of CPT-1 is inhibited by malonyl-CoA. The inhibition of ACC activity leads to a reduced malonyl-CoA production, resulting in the suppression of FFA synthesis and, reciprocally, the enhancement of FFA β-oxidation [33]. In our experiment, mangiferin increased significantly CPT-1 protein expression and inhibited ACC activity in liver of hyperlipidemic rats and in OA induced HepG2 cells, indicating that mangiferin could significantly increase fatty acid β-oxidation. DGAT2 is a rate-limiting enzyme that catalyzes the final step in TG synthesis by facilitating the linkage of diacylglycerol with a long chain fatty acyl-CoA [25]. Mangiferin decreased the expression of DGAT2 protein in the liver of hyperlipidemic rats and in OA induced HepG2 cells, indicating that mangiferin could reduce the biosynthesis of intracellular TG from fatty acid. Taken together, these observations suggest that mangiferin promotes fatty acid β-oxidation instead of esterification in liver.

AMPK, an upstream regulator of CD36, CPT1, ACC and DGAT2, is a critical enzyme involved in FFA metabolism [26]. It plays an important role in lipid metabolism and FFA oxidation in the liver [20]. Results from our study verified that the AMPK pathway mediates the effect of mangiferin on FFA metabolism toward an enhancement of fatty acid β-oxidation and inhibition of TG synthesis in liver of hyperlipidemic rats and in HepG2 cells. High ratio of AMP to ATP, and LKB1 as an upstream kinase, can activate AMPK. In this study, mangiferin increases the ratio of AMP to ATP in a dose-dependent manner, but has no effect on LKB1 phosphorylation, indicating that the effect of mangiferin on AMPK is accomplished through its effect on the ratio of AMP to ATP. However, the mechanism of how mangiferin causes an increase in the ratio of AMP to ATP needs to be studied further.

In conclusion, we investigated systematically the effect of mangiferin on FFA levels and FFA metabolism, and its possible mechanism for the first time. Mangiferin decreases the levels of FFA in both plasma and liver tissue in hyperlipidemic rats, promotes FFA catabolism by regulating the key enzymes of FFA uptake and utilization and inhibiting intracellular TG synthesis in liver. The effect of mangiferin on decreasing plasma FFA levels and increasing FFA catabolism is possibly exerted through the AMPK pathway by enhancing the ratio of AMP to ATP. Therefore, mangiferin results in beneficial effects on FFA metabolism, which may improve metabolic syndrome.

## Materials and Methods

### Animals

All rats were purchased from Shanghai SLAC Laboratory. Animals were housed in cages individually in an environmentally controlled room at 21±2.0°C and 50±5% humidity. Animals were subject to a 12∶12 h light∶dark cycle and had access to food and water ad libitum. After an acclimatization period of one week, the animals were used in the following experiments.

#### Experiment I

Forty male rats and forty female rats (100±20 g) were divided into 4 groups respectively, 10 rats in each group, treated with different concentrations of mangiferin (0, 100, 200, 400 mg/kg BW) by oral gavage for 30 days. The blood chemistry results, hematologic measures, and liver enzyme values were determined by using SYSMEX-SF-3000 Automatic Hematology Analyzer (Sysmex corporation, Kobe, Japan).

#### Experiment II

Sixty male rats (220±20 g) were divided randomly into six groups (n = 10 in each group): control group, fed an AIN-93G diet; hyperlipidemia group, fed a high-fat diet (48.1% of energy derived from fat, 34.2% from carbohydrates, and 17.7% from protein); positive drug group, fed a high-fat diet and fenofibrate at dose of 100 mg/kg BW/d; mangiferin-supplemented groups, fed the high-fat diet and different doses of mangiferin (50, 100, 150 mg/kg BW/d). Mangiferin (>90%, HPLC; Tianjin zhongxin pharmaceutical Group Co Ltd, Tianjin, China) and fenofibrate was given by oral gavage in 1% carboxymethyl cellulose buffer solution. Rats in the control and hyperlipidemia groups were given 1% carboxymethyl cellulose buffer only. After 6 weeks of mangiferin supplementation, all the animals were fasted for 12 h until sacrifice. Rats were anesthetized by pentobarbital sodium and blood samples were taken from abdominal aorta into EDTA-tubes. Abdominal and epididymal fat pads were removed and weighted separately. Liver was collected and stored at −80°C for analysis. The experimental protocols were approved by the Institutional Animal Care and Use Committee of Harbin Medical University, and conducted in compliance with the animal-use guidelines (SYXK (Hei) 2006-010).

### Reagents

Dulbecco's Modified Eagle Medium (DMEM) was purchased from GIBCO (Grand Island, NY). Fetal bovine serum (FBS) was purchased from Sijiqing Co (Hangzhou, China). Mangiferin for cell experiment, oleic acid (OA), fenofibrate and compound C were obtained from Sigma-Aldrich (St. Louis, MO). Mangiferin (90%, HPLC) for animal experiment were obtained from Zhongxin Innova Laboratories (Tianjin, China). Mangiferin, fenofibrate and compound C were dissolved in dimethyl sulfoxide (DMSO) and diluted with culture medium. Antibodies against AMP-activated protein kinase α (AMPKα), phosphor-AMPKα (p-AMPKα), acetyl CoA carboxylase (ACC) and phosphor-ACC (p-ACC) were purchased from Cell Signaling (Beverly, MA). Antibody against fatty acid translocase (CD36) was purchased from Cayman Chemical (Ann Arbor, MI). Antibodies against LKB1, phosphor-LKB1, carnitine palmitoyltransferase 1 (CPT-1), acyl-CoA: diacylglycerol acyltransferase-2 (DGAT2), GAPDH and β-actin were purchased from Santa Cruz Biotechnology (Santa Cruz, CA).

### Cell culture and treatment

The HepG2 cell line was obtained from the Chinese Academy of Science (Shanghai, China). The cells were maintained in DMEM containing 10% (v/v) FBS and 1% antibiotic/antimycotic at 37°C in an atmosphere containing 95% air and 5% CO_2_. The cytotoxicity of mangiferin was determined by the 3-(4, 5-Dimethylthiazol-2-yl)-2, 5-diphenyltetrazolium bromide (MTT) assay. When 70–80% confluence was reached, the cells were treated with different concentrations of mangiferin (0, 12.5, 25, 50, 100 µmol/L) or 100 µmol/L fenofibrate (positive control) in serum-free medium with 1% FFA-free bovine serum albumin (BSA) in the presence of OA (0.2 mmol/L) for 24 h. In parallel, HepG2 cells treated with an equivalent volume of DMSO (0.1%) without OA were used as the normal control. HepG2 cells were treated with OA (0.2 mmol/L) in order to simulate the condition of high level of plasma FFA in liver cells. Compound C (25 µmol/L) was added to HepG2 cells 1 h before treatment with mangiferin to verify the AMPK signal pathway.

### Determination of total cholesterol (TC), TG, FFA and β-hydroxybutyrate in plasma and liver tissue

A portion of liver was homogenized and the lipids were extracted with Chloroform and methanol (1∶2 v/v) 2 times as described by Bligh et al [15], and the supernatant were collected for the determination of TC, TG and FFA. TC and TG were determined by enzymatic colorimetric methods with commercial kits (Zhongsheng, Beijing, China), FFA was determined by GC-MS(TRACE GC/PolarisQ MS, Thermo Finnigan, USA) as described previously by our laboratory [16]. β-hydroxybutyrate concentrations in blood and liver were spectrophotometrically assayed by commercial test kits (Co-health Ltd, Beijing, China) [17].

### Determinations of intracellular TG, intracellular OA and OA uptake in medium in HepG2 cells

HepG2 cells were incubated with 0.2 mmol/L OA alone, or with different concentrations of mangiferin (12.5, 25, 50, 100 µmol/L) or 100 µmol/L fenofibrate simultaneously in serum-free medium with 1% FFA-free BSA for 24 h. The cellular lipid was extracted using a previously described method [18]. Cell protein was determined by the Bradford method. The intracellular TG mass was quantified spectrophotometrically at 490 nm using a TG test kit (Zhongsheng, Beijing, China). The experiments were repeated three times.

The concentration of OA remaining in the medium after treatment was determined to assess the uptake of OA by HepG2 cells. OA was measured by GC-MS (TRACE GC/PolarisQ MS, Thermo Finnigan, USA). Aliquots (1 mL) of cell culture medium, or 200 µL of cell lysates in each group were spiked with an internal standard (IS) working solution (200 µL heptadecanoic acid C17:0, 200 µg/mL), and the following method of sample pretreatment and GC-MS conditions was used, as described previously by our laboratory [16].

### Western blot analysis for the key proteins and enzymes of FFA metabolism

The proteins CD36, CPT-1, ACC, DGAT2, AMPK and LKB1 were determined by western blot analysis. Proteins from the cultured cells or liver tissues were extracted with a RIPA lysis buffer (50 mmol/L Tris, pH 7.4, 150 mmol/L NaCl, 1% Triton X-100, 1% sodium deoxycholate, 0.1% SDS, 1 mg/mL leupeptin, 50 mmol/L sodium fluoride, 1 mmol/L sodium orthovanadate, 1 mmol/L phenylmethylsulfonyl fluoride). Protein concentrations were determined by the Bradford method. Equal amounts of protein were separated by SDS-PAGE, and electro-transferred onto polyvinylidene difluoride (PVDF) membranes. The membranes were blocked with 1% BSA and TBS-T (50 mmol/L Tris-HCl, pH 7.5, 150 mmol/L NaCl, 0.1% Tween 20) for 0.5 h at room temperature. The membranes were incubated overnight at 4°C with primary antibodies in TBS-T. The membranes were washed three times with TBS-T (6 minutes each), and incubated with appropriate secondary antibodies for 1 h at room temperature. The signal was amplified by color development using the ProtoBlot II AP System with a stabilized substrate (Promega Corporation, Madison, USA). Data were presented as the ratios of target protein to *β*-actin. For each study, western blot analysis was conducted three times and representative blots were shown.

### Measurements of intracellular AMP and ATP in HepG2 cells

After the treatment as described above, cells were lysed in 300 µ L media with 20 µ L of 1 mol/L HClO4. HClO4 was removed by mixed phase extraction employing tri-n-octylamine and Freon 11 (11.75∶13.25; v/v) [19]. Lysates were analzyed by HPLC on a Waters C18 column, using a Waters 2695 Separations Module and a 2487 Dual Absorbance Detector (Waters Corporation, USA). The HPLC system consisted of 0.1 mol/L potassium phosphate buffer (contain 3 mmol/L tetrabutylammonium hydrogen, pH = 5.85) and methanol (88∶12) as the mobile phase, with the detection wavelength at 254 nm, and the flow rate at 1 mL/min. Experiments were repeated three times.

### Statistical analysis

All data were analyzed for statistical significance with SPSS 13.0 software. Data were presented as means ± standard deviation. Statistical analysis used one-way ANOVA. *P*<0.05 was considered to be statistically significant.

## Supporting Information

Table S1 **Effects of mangiferin on blood chemistry results, hematologic measures and liver enzyme values in 30-day feeding test.**
(DOC)

Table S2 **List of all actual numbers to generate the relative results of** **Figure 2** **.**
(DOC)
