# Supplementary material for: Mangiferin Decreases Plasma Free Fatty Acids through Promoting Its Catabolism in Liver by Activation of AMPK
Source: PLoS One. 2012 Jan 23;7(1):e30782. doi: 10.1371/journal.pone.0030782 (PMC3264633; doi:10.1371/journal.pone.0030782)
Supplement: Table S1 — Effects of mangiferin on blood chemistry results, hematologic measures and liver enzyme values in 30-day feeding test. (DOC) [file pone.0030782.s001.doc]

**Table S1** Effects of mangiferin on blood chemistry results, hematologic measures and liver enzyme values in normal rats

|  | **Mangiferin dose level (mg/kg bw)** | | | | | | | |
| --- | --- | --- | --- | --- | --- | --- | --- | --- |
|  | **Male (n=40, 10 each group)** | | | | **Female (n=40, 10 each group)** | | | |
|  | **0** | **100** | **200** | **400** | **0** | **100** | **200** | **400** |
| BW (g) | 276±27.9 | 258±28.7 | 270±29.8 | 264±29.8 | 227±38.2 | 221±31.2 | 232±31.4 | 217±30.9 |
| RBC (×10^12^/L) | 9.11±0.70 | 8.77±0.69 | 9.02±0.73 | 8.89±0.67 | 8.91±0.69 | 8.66±0.73 | 9.00±0.70 | 8.55±0.72 |
| WBC (×10^9^/L) | 14.7±1.66 | 13.8±1.39 | 14.5±1.93 | 14.2±1.36 | 13.4±1.44 | 14.7±1.96 | 14.2±1.62 | 13.7±1.41 |
| HGB (g/L) | 145±16.3 | 137±15.9 | 143±14.9 | 150±18.5 | 138±16.8 | 136±15.7 | 144±16.0 | 133±15.5 |
| ALT (U/L) | 26.5±6.6 | 25.9±6.0 | 27.8±8.6 | 24.2±7.9 | 26.6±8.2 | 27.3±7.0 | 25.3±8.7 | 28.8±7.8 |
| AST (U/L) | 28.1±8.6 | 28.4±8.2 | 26.6±9.1 | 27.9±9.4 | 29.1±8.3 | 27.3±7.4 | 28.2±8.0 | 27.7±8.6 |
| BUN (mmol/L) | 7.12±1.62 | 7.26±1.49 | 6.94±1.14 | 7.04±1.66 | 7.20±1.61 | 6.92±1.42 | 6.84±1.43 | 7.16±1.64 |
| CRE (umol/L) | 59.7±7.48 | 56.8±8.30 | 63.8±9.25 | 61.3±9.44 | 61.9±7.80 | 58.4±9.65 | 55.9±10.3 | 60.4±10.1 |
| Glu (mmol/L) | 5.64±1.15 | 5.87±1.38 | 6.12±1.07 | 5.59±1.21 | 5.44±1.00 | 6.04±1.29 | 5.89±1.26 | 5.66±1.01 |
| TC (mmol/L) | 2.64±0.36 | 2.44±0.40 | 2.51±0.37 | 2.42±0.38 | 2.76±0.41 | 2.79±038 | 2.53±0.42 | 2.55±0.43 |
| TG (mmol/L) | 1.23±0.14 | 1.20±0.15 | 1.22±0.14 | 1.17±0.15 | 1.26±0. 16 | 1.24±0.14 | 1.19±0.13 | 1.22±0.15 |

Data are means ± SD (n=10), the intervention had no significant effect on any variables by one-factor ANCOVA in normal rats treated with mangiferin for 30 days.

BW, body weight; RBC, red blood cell; WBC, white blood cell; HGB, hemoglobin; ALT, alanine aminotransferase; AST, aspartate aminotransferase; BUN, blood urea nitrogen; CRE, creatinine; Glu, glucose; TC, total cholesterol; TG, triglycerides.
