# Supplementary material for: Mangiferin Decreases Plasma Free Fatty Acids through Promoting Its Catabolism in Liver by Activation of AMPK
Source: PLoS One. 2012 Jan 23;7(1):e30782. doi: 10.1371/journal.pone.0030782 (PMC3264633; doi:10.1371/journal.pone.0030782)
Supplement: Table S2 — List of all actual numbers to generate the relative results of Figure 2 . (DOC) [file pone.0030782.s002.doc]

**Table S2** Effects of mangiferin on OA in medium, intracellular OA and intracellular TG in HepG2 cells

| **Measurement** | **OA in medium**  **(μg/ ml)** | **Intracellular OA**  **(μg/ mg cell protein)** | **Intracellular TG**  **(μg/ mg cell protein)** |
| --- | --- | --- | --- |
| Control | 15.29±2.35 | 407.6±57.4 | 137.6±17.1 |
| OA(0.2mmol//L) | 41.25±3.56^##^ | 1632.7±247.1^##^ | 233.2±24.4^#^ |
| OA(0.2mmol//L)+fenofibrate(100μmol/L) | 29.02±3.77^**^ | 1112.4±176.5^*^ | 143.2±19.8^**^ |
| OA(0.2mmol//L)+mangiferin(12.5μmol/L) | 39.30±4.18 | 1452.5±287.4 | 216.9±22.3 |
| OA(0.2mmol//L)+mangiferin(25μmol/L) | 33.81±3.87^*^ | 1387.8±185.2 | 207.5±19.7 |
| OA(0.2mmol//L)+mangiferin(50μmol/L) | 31.70±3.94^*^ | 1093.9±146.8^*^ | 172.6±21.2^*^ |
| OA(0.2mmol//L)+mangiferin(100μmol/L) | 27.86±3.32^**^ | 958.7±109.6^**^ | 160.3±20.7^*^ |

Data are means ± SD (n=3), ^#^ *P* < 0.05 ^##^ *P* < 0.01 indicate statistically significant differences when compared with control group. ^*^ *P* < 0.05 and ^**^ *P* < 0.01 indicate statistically significant differences when compared with OA group.
